# Ultrasonic Generation of Pulsatile and Sequential Therapeutic Delivery Profiles from Calcium-Crosslinked Alginate Hydrogels

**DOI:** 10.3390/molecules24061048

**Published:** 2019-03-16

**Authors:** Tania Emi, Kendra Michaud, Emma Orton, Grace Santilli, Catherine Linh, Meaghan O’Connell, Fatima Issa, Stephen Kennedy

**Affiliations:** 1 Department of Chemical Engineering, University of Rhode Island, Kingston, RI 02881, USA; taniatemi@uri.edu (T.E.); grace_santilli@my.uri.edu (G.S.); 2 Department of Electrical, Computer, and Biomedical Engineering, University of Rhode Island, Kingston, RI 028881, USA; k3ndranicole@my.uri.edu (K.M.); emma_orton@my.uri.edu (E.O.); catherine_linh@my.uri.edu (C.L.); moconnell2@my.uri.edu (M.O.); fatima_issa@my.uri.edu (F.I.)

**Keywords:** ultrasound, on-demand release, cancer therapy, drug delivery, biomaterials

## Abstract

Control over of biological processes can potentially be therapeutically regulated through localized biomolecular deliveries. While implantable hydrogels can provide localized therapeutic deliveries, they do not traditionally provide the temporally complex therapeutic delivery profiles required to regulate complex biological processes. Ionically crosslinked alginate hydrogels have been shown to release encapsulated payloads in response to a remotely applied ultrasonic stimulus, thus potentially enabling more temporally complex therapeutic delivery profiles. However, thorough characterizations of how different types of therapeutic payloads are retained and ultrasonically released need to be performed. Additionally, the impact of potentially disruptive ultrasonic stimulations on hydrogel structure and temperature need to be characterized to better understand what range of ultrasonic signals can be used to trigger release. To perform these characterizations, calcium-crosslinked alginate hydrogels were loaded with various model macromolecules (dextrans), chemotherapeutics, and protein signaling factors and exposed to a variety of single-pulse and multi-pulse ultrasonic signals at various amplitudes and durations. In response to single-pulsed ultrasonic exposures, quantifications of molecular release, degree of gel erosion, and increase in hydrogel temperature revealed that the ultrasonic stimulations required for statistically significant therapeutic deliveries often eroded and heated the gels to unacceptable levels. However, multi-pulse ultrasonic exposures were shown to achieve significant amounts of therapeutic release while keeping gel erosion and temperature increase at modest levels. Finally, experiments were performed demonstrating that ultrasonic stimulation could be used to generate drug release profiles shown to have potential therapeutic benefits (e.g., pulsatile and sequential anticancer delivery profiles). This work underscores the potential of using ultrasonically responsive polymeric hydrogels for providing on-demand control over more complex therapeutic deliver profiles and enhancing drug delivery strategies in cancer therapies and beyond.

## 1. Introduction

Biological systems conduct themselves with high degrees of spatial and temporal complexity. Many of the biological processes that underlie injury and disease constantly change in space and time and must be regulated with spatiotemporal precision for proper therapeutic outcome. For instance, wound healing and tissue engineering applications involve complex cell instructions where the spatial gradient, timing, and sequence of bioactive molecules are vital to orchestrating proper tissue regeneration [1,2,3,4,5]. Regeneration of tissues requires the generation of vascular networks. This involves initializing angiogenic sprouting from nearby existing vasculature (through the establishment of pro-angiogenic signaling factor gradients emanating away from the regeneration site). After these sprouts have invaded the site of regeneration, they must mature into larger, blood-perfusing vessels (through localized presentations of pro-maturation signaling factors) [1,2]. Regenerating tissues also demands the establishment of tissue-specific cells types, requiring recruitment of progenitor cells to the injury site (through the establishment of recruitment factor gradients emanating away from the injury site). Then, these recruited progenitors must be directed to proliferate and differentiate (through localized presentations of proliferation and differentiation signaling factors) [3,6,7,8,9]. Beyond regenerative therapies, cancer treatment strategies often aim to present anticancer therapeutics locally at tumor sites to minimize off-target side effects and polymeric hydrogel materials can be used to provide this localized delivery [10]. However, there is growing evidence that the localized and sustained therapeutic deliveries provided by hydrogels are not optimal and may be particularly not well-suited for preventing adaptive resistance. Many emerging tumor treatment strategies thus involve more temporally dynamic, pulsatile therapeutic delivery schedules [11,12,13] and even sequences of multiple anticancer therapeutics [14,15,16,17,18,19,20,21].

Polymeric hydrogels have shown the ability to release bioactive payloads in response to externally applied stimuli such as changes in pH [22], light [23], temperature [24], electric field [25], magnetic field [26,27,28,29,30,31], ultrasound [32,33,34,35]. These stimuli-responsive polymeric hydrogel systems could thus provide the localized, on-demand, complex therapeutic deliveries needed to direct spatiotemporally complex biological processes. Ultrasonically responsive hydrogels are of particular interest in that ultrasound (US) has been extensively used in a wide variety of therapeutic applications owing to its safety, relatively high spatiotemporal precision and ability to penetrate tissues [36,37,38]. Ca^2+^ crosslinked hydrogels respond to ultrasound stimulation reversibly (i.e., they can self-heal due to the calcium ions re-crosslinking the network after US-stimulation) which provides enhanced release when stimulated and minimal baseline release when not stimulated [33,39]. However, repeated and/or too-intense of US stimulus can irreversibly damage the hydrogel scaffold. This is undesirable in that (i) alteration of the hydrogel structure can impact baseline release kinetics and (ii) loss of hydrogel integrity could prohibit subsequent ultrasonically triggered release events. Additionally, repeated and/or too-intense of US stimulus can render sensitive payloads bio-inactive. While several previous studies have demonstrated ultrasonically triggered therapeutic release from hydrogels—with some systems even demonstrating the ability to produce sequential release profiles by leveraging multiple ultrasonically triggerable compartments [40,41,42]—it remains unclear (i) how the intensity and duration of ultrasonic stimulation impacts release for a diverse range of therapeutics, (ii) how the intensity and duration of ultrasonic stimulation impacts temperature increase and hydrogel integrity, and (iii) if pulsatile and multi-payload delivery profiles can be produced through ultrasonic stimulation of bulk hydrogel materials. The work presented here endeavored to elucidate these issues by quantifying the amount of molecular release (of four model macromolecules of various size and charge, three chemotherapeutics, and two protein-signaling factors) and the degree of gel erosion/heating after exposure to a variety of single-pulse ultrasonic signals (of various amplitude and duration) and multi-pulse signals (with different pulse numbers). Then, data obtained from these characterizations were used to design experiments that demonstrated the ability to ultrasonically produce pulsatile and sequential delivery profiles from these gel systems.

## 2. Results

### 2.1. Impact of Ultrasonic Intensity and Duration on Therapeutic Release from Ca^2+^ Crosslinked Hydrogels

**Figure 1:**
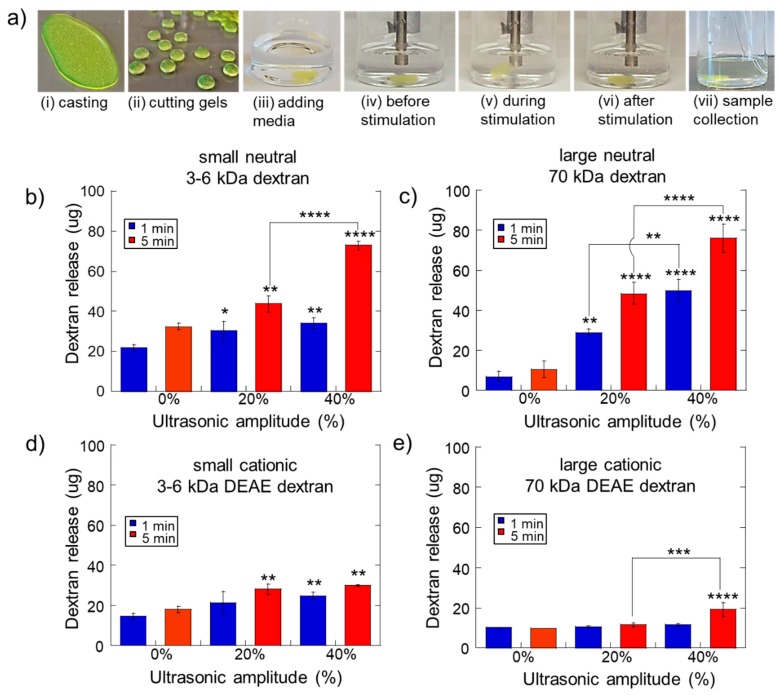
Ultrasonic amplitude, ultrasonic duration, molecular size, and molecular charge influence the amount of molecular release from Ca^2+^ crosslinked hydrogels. (**a**) FITC-dextran-loaded Ca^2+^ crosslinked alginate hydrogel fabrication: casting gel on a sigma-coated glass plate (i), cutting into 8-mm-diameter cylindrical gels using a biopsy punch (ii), putting a gel in a scintillation vials with 5 mL DPBS (iii), gels before, during, and after ultrasound stimulation (iv–vi), and 1 mL sample collection for quantifying molecular release (vii). (**b**–**e**) FITC-dextran release (**b**,**c**) and diethylaminoethyl (DEAE)-FITC-dextran release (**d**,**e**) vs. ultrasonic amplitude for gels exposed to 1-min (blue) and 5-min (red) of ultrasonic stimulation. *, **, ***, and **** indicate statistically significant differences with *p* < 0.05, 0.01, 0.001, and 0.0001, respectively, compared to controls (0% amplitude) unless otherwise specified with lines. N = 4.

To investigate how different ultrasonic exposures impacted therapeutic release from calcium-crosslinked hydrogels, 1 wt% alginate hydrogels with 30 mM calcium crosslinker were fabricated and pre-loaded with a variety of different molecules (dextrans of various size and charge, chemotherapeutic agents, and different protein signaling factors) (Figure 1a (i): hydrogels loaded with FITC-labeled dextran). This hydrogel formulation was previously reported to be capable of ultrasonically triggered drug delivery [33,39,42]. Immediately after making the hydrogels, they were cut into small cylinders and were transferred to scintillation vials with 5 mL Dulbecco’s phosphate buffered saline (DPBS) and subjected to ultrasound stimulation at various amplitudes for various durations (Figure 1a (ii)–(vi)). Immediately following ultrasonic stimulation, samples were collected to ascertain the amount of the molecular release (Figure 1a (vii)) (see Materials and Methods for more details).

We hypothesized that the size and charge of molecules would have an impact on ultrasonically stimulated release from these alginate hydrogels. To test this hypothesis, we studied the release of four different model macromolecules (FITC dextrans) when subjected to US stimulation of 0%, 20% and 40% amplitude for durations of 1 and 5 min. Two sizes were used: relatively small dextrans of 3–6 kDa and larger dextrans of 70 kDa. Additionally, to investigate the impact of charge, uncharged dextrans were used as well as diethylaminoethyl (DEAE)-modified cationic dextrans. It was thought that the positively charged side groups on the DEAE dextrans would electrostatically interact with the negatively charged groups presented by the alginate hydrogel matrix, thus impacting release. Indeed, cationic dextrans released at levels generally lower (Figure 1d,e) than their neutrally charged counterparts (Figure 1b,c). In some instances, the size of the dextran impacted release as well. For example, ultrasonic stimulation did not yield higher amounts of release than controls (0% amplitude) for hydrogels loaded with larger cationic dextran, except when stimulated at 40% amplitude for 5 min (Figure 1e). However, smaller cationic dextrans did release with statistical significance at 20% amplitude for 5 min, 40% amplitude for 1 min, and 40% amplitude for 5 min (Figure 1d). For neutrally charged dextrans, size seemed to particularly impact baseline, unstimulated release. For instance, higher amounts of smaller neutral dextran released under 0% ultrasonic amplitude (Figure 1b, blue and red bars at 0%) than larger neutral dextrans (Figure 1c, blue and red bars at 0%). These results are consistent with the notion that larger, more electrostatically interactive molecules exhibit less mobility in and through the negatively charged hydrogel matrix and thus exit the matrix at reduced rates.

As expected, the amplitude and duration of ultrasonic stimulation also influenced release. Generally, for increasing ultrasonic amplitudes, there was an upward trend in the amount of molecular release (Figure 1b,c: both blue and red bars trended upwards at increasing ultrasonic amplitudes). In fact, nearly all the drug contained in each hydrogel (80.4 µg/gel) was released when stimulating neutral dextran-loaded hydrogels with 40% ultrasonic amplitude for 5 min (Figure 1b,c, red bars at 40%). However, this trend was less pronounced when examining the cationic dextrans. The smaller cationic dextran maintained only a modest upward trend as ultrasonic amplitude was increased (Figure 1d). This upward trend was not exhibited well for the larger cationic dextran (Figure 1e). Thus, for larger molecules that exhibit the opposite charge as the polymer matrix, there seems less ability to ultrasonically select the amount of molecular release by stimulating at specific amplitudes and durations. While the larger cationic dextran did release from the hydrogels with statistical significance when stimulated at 40% ultrasonic amplitude for 5 min, these degrees of ultrasonic stimulation may be disruptive to both the hydrogel structure and the payload itself (see Section 2.2).

**Figure 2:**
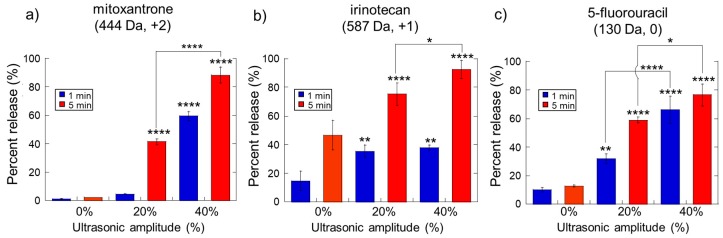
The amplitude and duration of ultrasonic stimulation impact the amount of chemotherapeutic released. Release vs. ultrasonic amplitude for mitoxantrone (**a**), irinotecan (**b**), and 5-fluorouracil (**c**). *, **, ***, and **** indicate statistically significant differences with *p* < 0.05, 0.01, 0.001, and 0.0001, respectively, compared to controls (0% amplitude) unless otherwise specified with lines. N = 4.

While the use of dextrans as model drugs was useful for understanding general trends in how molecular weight and charge impacted ultrasonically triggered delivery, we wanted to perform experiments characterizing the release of actual therapeutic agents. Therefore, we investigated the release of small molecular weight chemotherapeutics (mitoxantrone, irinotecan, and 5-fluorouracil) from these hydrogels under the ultrasonic stimulus. Note that when using different chemotherapeutic agents, different amounts of agent needed to be loaded in the hydrogels in order for the amount released to be detectable and quantifiable via absorbance spectroscopy (40.2 µg/gel, 201.1 µg/gel, and 80.4 µg/gel of chemotherapeutic for mitoxantrone, irinotecan, and 5FU respectively). Thus, these data are plotted as percent release on their y-axis rather than the amount released in weight for easier comparisons. As with the model dextrans, stronger US amplitudes generally resulted in a higher degree of drug release (Figure 2, bars trend upwards for increasing amounts of ultrasonic amplitude). Also, increasing the duration of ultrasonic stimulation increased the amount of drug release (Figure 2, red bars are generally higher than blue bars at the same ultrasonic amplitude).

The relative size and charge of these chemotherapeutics likely impacted release characteristics to some degree, though some inconsistencies were observed. Though all three-chemotherapeutics tested here were relatively small molecules, they did exhibit different charges: mitoxantrone (444.5 Da, +2 charge), irinotecan (586.7 Da, +1 charge), and 5FU (130.1 Da, neutral charge). Irinotecan diffused out of the gels at higher levels than mitoxantrone (Figure 2a,b: red and blue bars at 0% are higher for irinotecan than mitoxantrone). This seemed reasonable in that irinotecan had a lower positive charge density and would have a lower electrostatic affinity to the negatively charged alginate matrix. However, 5FU (which is neutral and the smallest chemotherapeutic examined here) did not diffuse out of the gels at lower amounts than irinotecan as initially expected (Figure 2b,c: bars at 0% US amplitude were, in fact, lower for 5FU than irinotecan). Note, however, that release kinetics are also based on parameters such as polarity. In fact, 5FU is polar and water-soluble (~1 g/L) whereas irinotecan is less water-soluble (~0.1 g/L). Thus, 5FU may present more opportunities to hydrogen-bond with alginate (which presents an abundance of electronegative groups) compared to the less polar irinotecan. But, for all three chemotherapeutics, application of ultrasound greatly enhanced release. 75%–90% of chemotherapeutic could be released when stimulated at 40% ultrasonic amplitude for 5 min (though with the caveat that this type of ultrasonic stimulation may be disruptive to the gel and possibly even the drug molecule itself).

**Figure 3:**
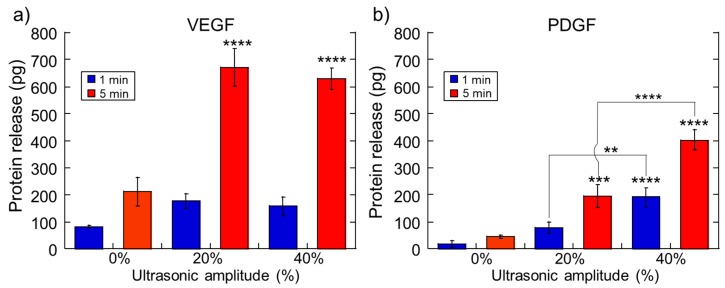
The amplitude and duration of ultrasonic stimulation impact the amount of signaling factor released. Release vs. ultrasonic intensity for Vascular Endothelial Growth Factor (VEGF) (**a**) and Platelet-Derived Growth Factor (PDGF) (**b**). *, **, ***, and **** indicate statistically significant differences with *p* < 0.05, 0.01, 0.001, and 0.0001, respectively, compared to controls (0% amplitude) unless otherwise specified with lines. N = 4.

To examine how ultrasonic stimulation impacted signaling factor release, calcium-crosslinked alginate gels were loaded with 6100 pg/gel of Vascular Endothelial Growth Factor (VEGF, a pro-angiogenic factor) and Platelet-Derived Growth Factor (PDGF, a pro-maturation factor) and stimulated at various ultrasonic amplitudes for different amounts of time. Despite being loaded with the same amount of protein, VEGF generally released in higher amounts than PDGF (Figure 3, comparing parts a to b). This is likely due to VEGF having a lower affinity to the alginate contained in the hydrogel’s matrix compared to PDGF’s alginate affinity. As was seen with dextrans and chemotherapeutics, longer ultrasonic durations and stronger amplitudes resulted in higher levels of protein release (Figure 3, upward trends at higher amplitudes and red bars being higher than blue bars at the same amplitude).

### 2.2. Erosion and Heating of Scaffold Due to Ultrasound Stimulation

**Figure 4:**
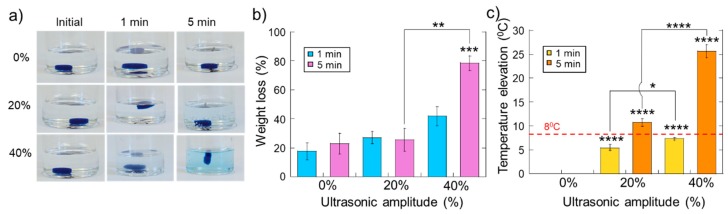
Ultrasound erodes and heats the gels in manners that are dependent on the intensity and duration of the applied ultrasound. (**a**) Photographs of mitoxantrone-loaded hydrogels after exposure to the US at the indicated amplitudes and durations. (**b**) Percent weight loss vs. US intensity for gels stimulated for 1 min (light blue) and 5 min (light purple). (**c**) Temperature elevation vs. US intensity for gels stimulated for 1 min (yellow) and 5 min (orange). *, **, ***, and **** indicate statistically significant differences with *p* < 0.05, 0.01, 0.001, and 0.0001, respectively, compared to controls (0% amplitude) unless otherwise specified with lines. N = 4.

While the data described in the previous section underscores the ability to ultrasonically regulate therapeutic release, application of ultrasonic stimulation can potentially irreversibly damage the hydrogel structure (prohibiting subsequent deliveries from the hydrogel) and/or reduce the bioactivity of sensitive therapeutic payloads (like protein signaling factors due to thermally induced conformational alterations). To investigate the range of ultrasonic stimulations that do not overly erode or heat the hydrogel structure, experiments were conducted that quantified the degree of hydrogel erosion and rise in temperature when exposed to different amplitudes and durations of ultrasound. As expected, with higher ultrasonic amplitudes and longer durations of exposure, hydrogel erosion was more pronounced (Figure 4a: photos of gels under different US conditions that were loaded with blue mitoxantrone). Quantifications of erosion (percent weight loss before/after ultrasound) revealed that the maximum US exposure (40% amplitude for 5 min) significantly eroded these gels (Figure 4b). However, all other US conditions yielded erosions lower than 50%. In terms of gel heating, all ultrasonic stimulations resulted in heating, as expected. However, it was desirable to keep temperature increases to under 8 °C (i.e., as to not increase temperatures from body temperature (37 °C) to temperatures that may impact protein conformation (45 °C)). Indeed, both the 1-min at 20% US amplitude and 5-min at 20% US amplitude conditions heated samples less than 8 °C (Figure 4c). When taking both gel erosion and heating into account, the 1-min 20% of US exposures appeared to be the safest to use. However, these 1-min, 20% US amplitude exposures did not yield statistically significant enhancements in release for cationic dextrans (Figure 1d,e, blue bars at 20%), mitoxantrone (Figure 2a, blue bar at 20%), or VEGF and PDGF (Figure 3, blue bars at 20%).

### 2.3. Triggered Therapeutic Delivery by Pulsing the Ultrasound Stimulation

Even though 1-min ultrasound exposures at 20% amplitude did not statistically enhance releases for a many of therapeutics tested here, we hypothesized that *repeated* 1-min, 20% US exposures could enhance therapeutic release while helping to preserve hydrogel structure and maintain relatively low temperatures. That is, the mechanism of ultrasonic release from these calcium-crosslinked alginate hydrogels is thought to be due to the displacement of calcium crosslinks (due to ultrasonically driven cavitation within the matrix) [33,39,41]. This displacement allows the matrix to unravel, and in the process, liberate drugs trapped within the matrix. If ultrasonically stimulated mildly enough, the matrix may remain relatively intact with enough free calcium crosslinkers present to re-crosslink the matrix upon termination of the ultrasonic stimulus (though some drug may be liberated from the matrix during stimulation). If the ultrasonic stimulation is of sufficient duration and intensity, the calcium crosslinked and the alginate polymer may become so dispersed that the matrix is not able to re-crosslink. So, if a gel is (i) initially stimulated with the US that does not overly disrupt the matrix but produces some drug release, then (ii) left to recover with the US is turned off, and then (iii) subsequently stimulated with US and allowed to recover, these pauses in ultrasonic stimulation may enable appreciable amounts of accumulated therapeutic release while providing time for the matrix to recover and for heat to dissipate between subsequent ultrasonic stimulations.

**Figure 5:**
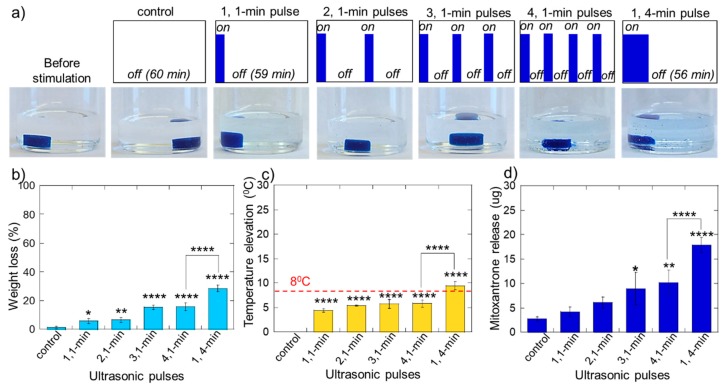
Pulsed ultrasound can enhance drug delivery while helping to control gel erosion and heating. (**a**) Photos of hydrogels (bottom row) after exposure to the illustrated ultrasonic pulse trains (top row). (**b**) Percent weight loss of hydrogels vs. pulse number. (**c**) Temperature rise vs. pulse number. (**d**) Mitoxantrone release vs. pulse number. *, **, ***, and **** indicate statistically significant differences with *p* < 0.05, 0.01, 0.001, and 0.0001, respectively, compared controls unless otherwise specified with lines. N = 4.

To test this hypothesis, we exposed calcium-crosslinked alginate gels to 1-min US pulses at 20% amplitude in pulse trains with varying pulse numbers over a 1-h period. Specifically, gels were exposed to controls (no pulses), one 1-min pulse, two 1-min pulses, three 1-min pulses, and four 1-min pulses (Figure 5a, moving left to right). Additionally, gels were exposed to one 4-min pulse (Figure 5a, rightmost illustration) which is equivalent to four 1-min pulses with no delay time between them. This was done to directly compare the same total exposure of ultrasonic stimulation with and without pauses (i.e., pulsed ultrasound vs. continuous). While increasing the number of ultrasonic pulses slightly increased the degree of erosion (Figure 5b: increasing values for 1, 2, 3, and 4 1-min pulses, though the 3 and 4 pulse conditions were statistically indifferent), temperature elevation did not appreciably increase for higher pulse numbers (Figure 5c: similar values for 1, 2, 3, and 4 1-min pulses). And, the amount of drug released did increase for increasing pulse numbers (Figure 5d: increasing values for 1, 2, 3, and 4 1-min pulses). Thus, increasing the number of ultrasonic pulses may provide a means to increase drug release while helping to minimize temperature increase and, to some extent, help maintain gel structure (compared to increasing the amplitude or duration of the US signal, which was shown to dramatically increase heating and gel erosion (Figure 4b,c).

Also of note was that one continuous 4-min pulse eroded and heated the gel much more than 4 individual 1-min pulses separated by 14 min (Figure 5a,b: comparing 1,4-min bars to 4, 4-min bars). While the highly eroded gel naturally yielded a higher degree of drug release (Figure 5c, comparing 1,4-min bars to 4, 4-min bars), these results suggest that pausing between shorter US pulses can help maintain the structure and temperature of the gel while still producing statistically significant amounts of drug release.

### 2.4. Ultrasonically Generating Pulsatile Therapeutic Delivery Profiles

**Figure 6:**
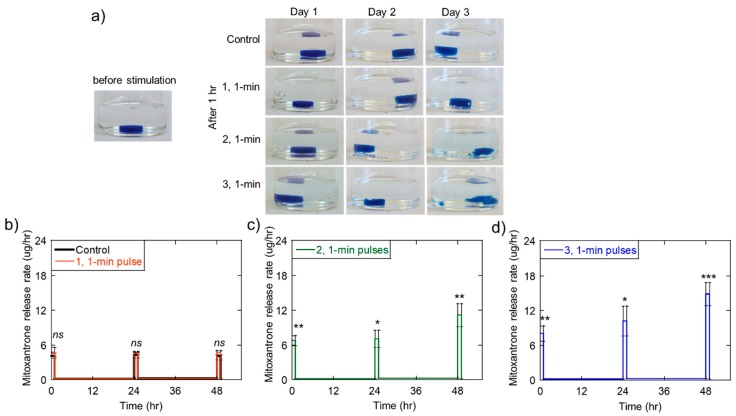
Ultrasound can be used to generate pulsatile chemotherapeutic delivery profiles over a 3-day period, though gel structure is not maintained, and delivery rates change vs. time. (**a**) Photos of hydrogels immediately after ultrasonic exposures on the indicated days. (**b**–**d**) The rate of mitoxantrone release vs. time when stimulated with pulsed ultrasound for 1 h each day for control and one 1-min pulse (**b**), two 1-min pulses (**c**), and three 1-min pulses (**d**). Plots of release rates vs. time are meant to highlight differences in release rates during periods of ultrasonic stimulation (from 0–1, 24–25, and 48–49 h) vs. periods of no stimulation (from 1–24 and 25–48 h). *, **, ***, and **** indicate statistically significant differences with *p* < 0.05, 0.01, 0.001, and 0.0001, respectively, relative to controls. n.s. indicates that no statistical significance was found compared to controls (*p* > 0.05). N = 4.

As discussed in the introduction, many emerging drug delivery strategies involve generating localized therapeutic delivery profiles that are more temporally complex. For example, there is growing evidence that pulsatile chemotherapeutic deliveries are more effective in destroying tumor cells than sustained deliveries [11,19,33,43]. Because of this potential, we aimed to demonstrate that ultrasonic stimulation could be used to generate pulsatile chemotherapeutic delivery profiles by periodically turning on and off the ultrasonic signal. To mimic a pulsatile mitoxantrone delivery profile that was previously demonstrated to enhance melanoma cell destruction [43], we exposed mitoxantrone-loaded, calcium-crosslinked alginate hydrogels to a 1-h period of pulsed US stimulation one time a day for 3 days. Specifically, for a 1-h period each day (for 3 days total), gels were stimulated with various US pulse trains composed of 1-min pulses at 20% amplitude: (i) 0 pulses (control), (ii) one 1-min pulse (and 59 min of no stimulation), (iii) two 1-min pulses (with 29 min of no stimulation following each pulse), and (iv) three 1-min pulses (with 19 min of no stimulation following each pulse). Photographs were taken immediately following each 1-h stimulation period to assess the relative degree of gel erosion (Figure 6a).

As expected, US exposure regiments that involved a higher number of pulses generally resulted in higher degrees of gel erosion. For US exposures of two and three 1-min US exposures, gels exhibited high degrees of erosion by the 3rd day of the experiment (Figure 6a, rightmost column, bottom two rows: gels began to fragment). While exposures to single 1-min US pulses allowed the gels to remain relatively intact (Figure 6a, rightmost column, 2nd from top row), these mild ultrasonic stimulations did not produce pulsed mitoxantrone release rates that were statistically higher than controls (Figure 6b, red and black curves are statistically similar). Note that the plots in Figure 6b through 6d are plots of mitoxantrone release rate vs. time, highlighting the difference in release rates during 1-h ultrasonic stimulation periods (from 0–1 h, 24–25 h, and 48–49 h) to release rates during periods of no ultrasonic stimulation (from 1–24 h and 25–48 h). The two and three 1-min pulsed US exposures did yield statistically significant mitoxantrone release rates during these 1-h “on” periods (Figure 6c,d) but did so in an inconsistent manner vs. time. That is, mitoxantrone release rates during 1-h “on” periods tended to increase over time, particularly on the final day of the experiments (Figure 6c,d: the pulse heights from 48–49 h were higher than from 0-1 and 24-25 h). We attributed this to the gel being more highly eroded from repeated ultrasonic exposures, having more surface area by day 3, and possibly being more fragile. Nonetheless, statistically significant pulsatile delivery rates were achieved, and the gels were not completely annihilated after these 3-day experiments.

**Figure 7:**
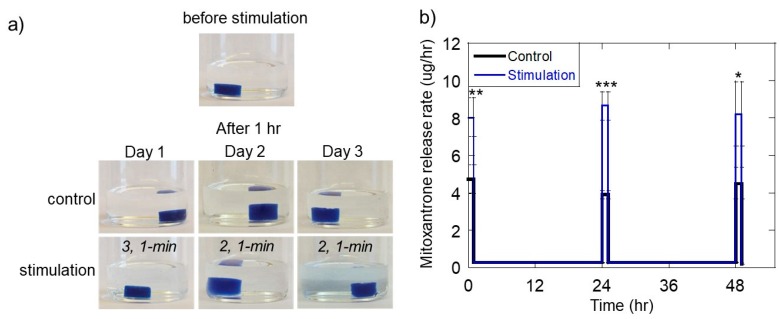
Pulsatile delivery profiles with consistent release rates vs. time can be achieved by altering the US pulse number over time. (**a**) Photos of hydrogels immediately after ultrasonic exposure on each day for control gels and those exposed to the indicated pulsed US regiment. (**b**) Release rate vs. time when stimulated with 3, 1-min; 2, 1-min; and 2, 1-min ultrasound pulses on day 1, 2, and 3, respectively. *, **, ***, and **** indicate statistically significant differences with *p* < 0.05, 0.01, 0.001, and 0.0001, respectively, relative to controls. N = 4.

To achieve more consistent pulsatile mitoxantrone delivery rates during subsequent “on” periods and to help maintain gel structure, we explored the idea of modifying US exposures vs. time. Specifically, we reduced the number of US pulses used during the 1-h US exposure period on day 2. That is, we used 3, 1-min pulses on day 1 (from 0–1 h), and then reduced the US pulse number to 2, 1-min pulses on days 2 and 3 (from 24–25 h and from 48–49 h). This strategy resulted in more mild gel erosion over time upon visual inspection (Figure 7a, bottom row) and provided more uniform mitoxantrone pulsed release rates during the 1-h “on” periods on subsequent days (Figure 7b, pulse heights of the blue curve are of similar heights at times 0, 24, and 48 h).

### 2.5. Sequential 5-Fluorouracil and Irinotecan Delivery On Melanoma Cells in Vitro

Some treatment strategies involve the sequential delivery of two or more therapeutics. For example, sequential delivery of 5FU and irinotecan is commonly used for treating colorectal cancer, though there is some debate as to which therapeutic should be delivered first: 5FU followed by irinotecan vs. irinotecan followed by 5FU [17,18,21]. Though particular sequences may be used to optimize therapeutic outcome, other parameters likely impact outcome as well: the duration of each therapeutic delivery, the temporal profile of each therapeutic delivery, the relative dose of each therapeutic, the spacing between each delivery, etc. For example, we conducted in vitro experiments on melanoma cells by exposing them to different delivery schedules involving both 5FU and irinotecan. B16F10 mouse melanoma cells were plated, allowed to grow for 36 h, and then exposed to different delivery schedules of 5FU and irinotecan for 36 h (Figure S1a, schedules s1–s4). B16F10 populations were monitored in real time using an xCELLigence system (which uses the electrical impedance of a well-plates surface to quantify cell population vs. time). xCELLigence data from these experiments are included as cell index (a measure of cell population) vs. time in Figure S1b. For the four therapeutic delivery schedules used here (s1–s4), all resulted in statistically similar B16f10 populations except for schedule s3 (Figure S1b, s3 purple curve resulted in statistically smaller populations at +36 h). So, for these particular 5FU and irinotecan doses, durations, and timings on this particular cell type in vitro, schedule s3 (5FU followed by irinotecan) was most effective in reducing melanoma cell populations. Critically, it is unclear what sequence would be optimal when testing a more diverse array of drug types, timings, doses, and sequences on different types of cancers in vivo. Therefore, material systems are urgently needed where these types of parameters can be readily controlled and modified from experiment to experiment. Ultrasonically responsive hydrogels could provide a material system for performing such optimizations in vivo if they can be tailored to generate multi-therapeutic, sequential delivery profiles.

### 2.6. Generation of the Sequential 5-Fluorouracil and Irinotecan Deliveries

To demonstrate the ability to produce sequential therapeutic delivery profiles from these ultrasonically responsive hydrogels, we aimed to mimic the delivery profile which reduced melanoma cell populations most efficiently in the above-described studies: schedule s3 (Figure S1a: initial 5FU delivery followed by irinotecan delivery starting at 18 h). So, one criteria of our design demanded that 5FU exit the gels rapidly at early time points. While 5FU did not diffusively release as much as irinotecan (comparing Figure 2b,c at 0% US amplitude), a non-trivial amount of 5FU still did released when no ultrasonic stimulation was applied (Figure 2c, ~10% release in 1 to 5 min). We believed that, over the course of 18 h, this amount of release could be therapeutically relevant. Thus, our strategy was to design the gel to initially release 5FU via diffusion with no ultrasonic stimulation and then ultrasonically trigger irinotecan release at 18 h. Additionally, the initial release of 5FU could be further enhanced by the method of loading 5FU into the gel. That is, instead of incorporating 5FU into the matrix during gel fabrication, 5FU could be loaded by soaking the gel in concentrated 5FU to the gel after gel fabrication. This would potentially limit how well 5FU incorporated into the gel and help it release more readily from the matrix.

To achieve delayed, ultrasonically triggered irinotecan delivery, it was desirable to have the gel release as little irinotecan as possible when no ultrasound was applied for the first 18 h and then release as much as possible when ultrasonically stimulated (at 18 h). However, examination of Figure 2b reveals that irinotecan releases at high rates, even when not ultrasonically stimulated (> 40% release in 5 min = 80 μg in 5 min = 16 μg/min). To enhance unstimulated irinotecan retention, we pursued two strategies. First, we believed that reducing the hydrogel’s mesh size (by increasing the polymer and crosslinker concentrations from 1.0 wt% alginate with 30 mM Ca^2+^ to 1.6 wt% alginate w/ith 50 mM Ca^2+^) would serve to better entrap and impede the release of irinotecan. This, of course, could also lead to lower amounts of release when ultrasonically stimulated. However, ultrasonically stimulated delivery of irinotecan could be very efficient (Figure 2b, 40% US amplitude for 5 min results in 90-100% release). So, we hoped that ultrasonic stimulation would still produce strong amounts of irinotecan release even with higher polymer and crosslinker concentrations. Second, we believed that rinsing the gels could help reduce the amount of diffusive irinotecan release at early time points. The studies presented in Figure 2 were performed immediately after gel formation and did not include a rinsing step. If they were rinsed, some of the less well-incorporated irinotecan could be removed, thereby reducing initial, diffusive release.

**Figure 8:**
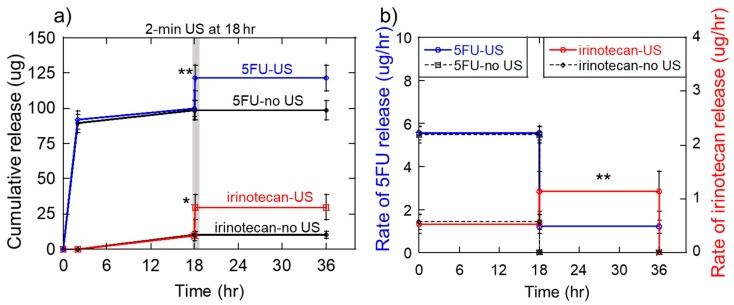
Ultrasonically responsive gels can be used to generate a triggered release of irinotecan after an initial burst release of 5-fluorouracil. (**a**) Cumulative release of 5-fluorouracil (blue) and irinotecan (red) vs time when ultrasonically stimulated for 2-min at 40% US amplitude at 18 h. Control gels (black) were not subjected to ultrasonic stimulation at 18 h. (**b**) The 18-h average rate of 5-fluorouracil and irinotecan release vs. time for the same conditions presented in part (a). * and ** indicate statistically significant differences with *p* < 0.05 and 0.01, respectively, relative to controls. N = 4.

Taking this all together, we produced 1.6 wt% alginate, 50 mM calcium-crosslinked gels (60% polymer and crosslinker) that were loaded with 200 μg/gel of irinotecan and 0 μg of 5FU. Gels were rinsed for 5 min in 5 mL of DPBS to remove excess irinotecan that did not incorporate well into the hydrogel matrix. Then, gels were soaked in 100 μL of concentrated 5FU (2 mg/mL) for 30 min to load the gels with 5FU in a manner that would be conducive to rapid, burst release. When placed in experimental media at time 0, indeed, gels released 5FU rapidly with low levels of irinotecan release (Figure 8a: 5FU curve (blue) rises to ~90 μg in the first 2 h whereas the irinotecan curve (red) does not rise to detectable levels). For the 18 h prior to ultrasonic stimulation, 5FU reached ~100 μg of cumulative release whereas irinotecan reached only ~10 μg (Figure 8a: values of blue and red curves at 18 h, respectively). Immediately after being stimulated with an ultrasonic signal at 40% US amplitude for 2 min, irinotecan released reached over 25 μg of cumulative release (Figure 8a, jump in the red curve just after 18 h). However, because the gel still had 5FU loaded in it, 5FU levels also increased in response to ultrasonic stimulation (Figure 8a, jump in a blue curve just after 18 h). For the remaining 18 h of the experiments, very little changes in 5FU and irinotecan release were observed (Figure 8a: flatline curves from just after US stimulation to 36 h). When looking at time-average release rates for the 18 h prior to and following the US, as designed, release rates of 5FU were higher before the US and fell to lower levels after US (Figure 8b, the blue curve is at higher values before 18 h). And, irinotecan release rates were lower before the US and increased after the US (Figure 8b: red curve is at higher values after ultrasound).

Erosion analysis of these studies revealed that the 2-min, 40% ultrasonic exposures applied to the gels at 18 h significantly eroded the gels (see photographs in Supplementary Information, Figure S2). This was not surprising I that 1-min ultrasonic exposures at 40% amplitude resulted in 40% gel erosion and 5-min exposures at 40% resulted in 80% erosion (Figure 4b, erosion values at 40% amplitude). Additionally, this was not viewed as problematic since no additional drug delivery was required after the 18-h time point (meaning the gel could have been completely annihilated between 18 h and 36 h).

## 3. Discussion

The ultrasonically responsive Ca^2+^-crosslinked hydrogels explored here were capable of producing various therapeutic delivery profiles that may provide benefits, particularly in cancer treatment strategies. The pulsatile chemotherapeutic release profiles demonstrated here (one, 1-h “on” period of enhanced mitoxantrone delivery per day for 3 days) were demonstrated to reduce melanoma cell populations more effectively than the more constant delivery profiles provided by traditional hydrogel materials [33,43]. It is thought that pulsatile deliveries can also help combat adaptive resistance in tumors [11,12,13,19]. Critically, the specific pulsatile delivery parameters (i.e., pulse height (dose), pulse width (duration), duty cycle (the relatively time when the delivery is high vs. low), and pulse number (how many periods of enhanced delivery per day)) that lead to optimized tumor cell destruction likely depends on the specific therapeutics used, the type of tumor, and other patient specifics. Though this presents a wide parametric space for optimization, ultrasonically responsive gels such as those presented here could help streamline these optimizations. That is, delivery parameters such as pulse height, width, and duty cycle can be flexibly regulated by altering the timing, duration, amplitude, and pulse number of the ultrasonic signal. The studies presented here have uncovered some additional considerations moving forward. Namely, that undesirable temperature increases and gel erosion occur at longer and higher-amplitude ultrasonic stimulations and that erosion can impact release kinetics vs. time (higher release rates vs. time). However, we presented strategies for dealing with these issues (i.e., by pulsing the ultrasonic signal, the gel is provided time to re-crosslink and the heat is allowed to dissipate between subsequent ultrasonic exposures).

While the pulsatile therapeutic delivery profiles generated in these studies (Figure 7b) are very similar to those demonstrated to improve melanoma cell destruction [43], it remains unclear with what level of flexibility ultrasonically responsive gels can produce pulsatile delivery profiles while preserving gel integrity, relatively low temperatures, and the viability of nearby cells and tissues. For example, studies are still needed to demonstrate how many subsequent pulses can be administered before the gel is either depleted of drug or erodes to the point of disused. Additionally, cell and tissue exposure studies under these same ultrasonic signals need to be performed to ensure safe operation in vivo. While studies do need to be performed to assess the safety of the specific ultrasonic signals used here (<2 W signals with exposure times on the order of minutes), it is known that ultrasonic signals an order of magnitude more powerful are needed to damage unwanted tissues (i.e., 30–38 W signals for treating glaucoma [44]). Indeed, the heating and disruption associated with ultrasonic stimulation in concert with chemotherapeutic delivery can have a synergistic effect when treating solid tumors, as demonstrated previously by Heubsch et al. [33]. However, when drug delivery treatments are not intended to destroy unwanted tissues, generation of heat is undesirable as it can harm nearby cells and tissues and cause denaturization of sensitive (i.e., protein-based) therapeutics. In these instances, irreversible protein denaturation starts at around 45 °C [45,46] (i.e., 8 °C above body temperature). This same temperature threshold of 45 °C is also associated with loss of viability for a number of cell types [45,46]. This is why our studies strived to identify ultrasonic stimulation conditions that resulted in temperature increase of less than 8 °C (Figure 4c and Figure 5b). In fact, pulsed ultrasound enabled statistically significant amounts of therapeutic release while keeping temperature increased under 8 °C (Figure 5c,d, both the 3 and 4, 1-min pulsed US conditions).

The studies conducted here have shown that 8 to 10 μg/min mitoxantrone pulses can be achieved while preserving gel structures for 3 days, but we have not explored pulse numbers beyond 3, ultrasonic amplitudes and durations beyond 20% and 1-min, or the pulsatile delivery of other anticancer payloads. Some fine-tuning can likely be achieved in terms of what types of ultrasonic signals are optimal. It is worth noting that the ultrasonic equipment used in these studies were inherently not optimized for this application (convention laboratory ultrasonic probes were used out of convenience). A wide variety of ultrasonic stimulation devices might be more effectively employed here. For example, ultrasonic arrays could be potentially employed to accurately target the hydrogel implant deep within tissues. Additionally, these hydrogels could be integrated into implantable devices where remotely controlled micro-ultrasonic sources would be used to locally stimulate the gels. This approach could provide several advantages: for example, (i) more localized gel stimulations may prevent damage to nearby cells and tissues and (ii) micro-US sources could be arrayed, providing more selectivity in terms of what therapeutic is being delivered at what time and in what direction. As is such, we believe these current studies underscore the potential of these ultrasonically responsive gels to provide pulsatile therapeutic deliveries and reinforce the need for continued exploration of their potential.

In addition to pulsatile chemotherapeutic deliveries, these ultrasonically responsive gels were demonstrated to produce a particular sequence of 5FU and irinotecan deliveries that were shown to reduce melanoma cells populations in vitro (i.e., 18 h of heightened 5FU delivery followed by 18 h of heightened irinotecan delivery) (Figure S1 and Figure 8). While a specific drug-loading strategy was used to attain this specific sequence (i.e., irinotecan was loaded during gel fabrication and rinsed for enhanced retention whereas 5FU was loaded just prior to the start of the experiment for enhanced burst release), similar strategies can be employed for achieving different sequences. For example, if—as some believe it to be more beneficial [17,21,47]—a sequence of irinotecan followed by 5FU is desired, then 5FU can be incorporated during gel formation and irinotecan can be added just prior to experimentation. Future work on the gel system demonstrated here includes optimizing parameters for achieving specific delivery rates at different times. For example, characterizations are needed to determine how hydrogel formulation (polymer and crosslinker concentrations), the ultrasonic stimulation parameters (US amplitude, US pulse width, US pulse number and delay between pulses), and the manner of loading various payloads impact the delivery rates of unstimulated irinotecan release rates and ultrasonically triggered irinotecan release rates. Additionally, future work needs to explore how changing these parameters can be used to control the timing of the sequence release. For instance, in these studies, delayed irinotecan release occurred at 18 h. Being able to flexibly control this time point will be critical to optimizing therapies. Ultrasonically responsive gel systems may provide unique advantages for optimizing the timing of therapeutic deliveries in that trigged deliveries directly correspond to the timing of ultrasonic signal application. Finally, some cancer treatment strategies utilize sequences of different therapeutics [14,15,16]. Continued exploration of these ultrasonically responsive gels are needed to determine if they are suitable for coordinating sequential delivery of other therapeutic agents in cancer treatment and beyond.

Finally, while these hydrogels demonstrated promise for producing sequential deliveries in cancer treatments, they may also be of use in treating injuries and diseases for which sequential biological process must be controlled. The ability to generate sequential delivery profiles would be of great utility in tissue engineering applications, where sequences of biological events (such as recruitment, proliferation, and differentiation) can be directed through sequential delivery of recruitment, proliferation, and differentiation signaling factors [3,6,7,8,9]. In addition to sequencing these factors (to establish tissue-specific cell types at the site of regeneration), vascularizing regenerated tissue is also critical. Generation of new vasculature also involves an important sequence of biological events: (i) generation of angiogenic sprouts from nearby, existing vasculature (which can be induced by projecting a pro-angiogenic signal gradients—such as VEGF—emanating away from the regeneration site) and (ii) maturation of the newly established network of angiogenic sprouts into larger, blood-perfusing vessels (which can directed through localized deliveries of pro-maturation factors—such as PDGF) [1,2]. Thus, calcium-crosslinked alginate gels may enable the establishment of VEGF gradients through diffusive release of VEGF followed by delayed, ultrasonically triggered release of PDGF. This may help optimize deliver strategies for regenerating vasculature in tissue engineering applications. In fact, we demonstrated VEGF releases in modest amounts under no ultrasonic stimulation (Figure 3a, 200 pg in 5 min at 0% US amplitude). This may aid in generating initial burst release of VEGF. PDGF tends to release in lower quantities when not ultrasonically stimulated (Figure 3b, ~50 pg in 5 min at 0% US amplitude). However, when ultrasonically stimulated, PDGF release can be substantially enhanced (for example, Figure 3b: ~200 pg in 1 min at 40% US amplitude). These attributes may help retain PDGF when not ultrasonically stimulated and enable its triggered release when stimulated.

## 4. Materials and Methods

### 4.1. Materials

B16F10 mouse melanoma cancer cells were purchased from American Type Culture Collection (ATCC, Manassas, VA, USA). Sodium alginate (Protanal LF20/40) of high molecular weight (~250 kDa) was donated by FMC BioPolymers (Philadelphia, PA, USA). Trypan Blue, Dulbecco’s modified eagle’s medium (DMEM), bovine serum albumin (BSA), fetal bovine serum (FBS), penicillin-streptomycin, and trypsin-EDTA solutions, morpholineethanesulfonic acid (MES) hydrate, Dulbecco’s phosphate buffered saline (DPBS), Sigmacote, activated charcoal, calcium sulfate dihydrate, irinotecan hydrochloride, 5-fluorouracil, mitoxantrone hydrochloride, fluorescein isothiocyanate (FITC) dextran (3–6 kDa and 70 kDa), fluorescein isothiocyanate diethylaminoethyl (FITC-DEAE) dextran (3–6 kDa and 70 kDa) and rhodamine dextran (10 kDa) were all purchased from Sigma-Aldrich (St. Louis, MO, USA). Vascular endothelial growth factor (VEGF) and Platelet-derived growth factor (PDGF) proteins and VEGF- and PDGF-DuoSet ELISA kits were purchased from R&D Systems, Inc (Minneapolis, MN, USA). 16-well xCELLigence e-plates were purchased from ACEA Biosciences, Inc. (San Diego, CA, USA).

### 4.2. Calcium Crosslinked Hydrogel Fabrication

To make ultrasonically responsive hydrogels, alginate was purified through dialysis (3500 MW cut off, Spectrum Laboratories, Compton, CA, USA), activated charcoal treatment, filtration, and lyophilization. In a previously described manner [33], alginate was dissolved in MES buffer (pH = 6.5) to make a 2.5 wt% alginate solution. 2 mL of 2.5 wt% alginate solution was mixed with 2 mL solution of a molecules of interest (drug, protein, or dextran dissolved in MES buffer). This 4 mL solution of alginate and “drug” was quickly mixed with 1 mL of 21 mg/mL CaSO_4_ in water slurry by linking two 5 mL syringes (Becton, Dickinson and Company, Franklin Lakes, NJ, USA) together with a LuerLock Connector (W. W. Granger Inc., Lake Forest, IL, USA) and rapidly transferring the mixture back and forth between syringes. Hydrogels were cast by quickly discharging the contents of the LuerLock syringes on glass plates and immediately placing a second glass plate on top of the gel mixture with 2-mm spacers in between the two plates. After gelation (30 min), individual 8 mm diameter cylindrical gels were cut using a biopsy punch. The resulting Ca^2+^ crosslinked 1 wt% alginate, 30 mM calcium-crosslinked hydrogels had 8 × 2 mm cylindrical structures.

### 4.3. Release Studies

The Ca^2+^ crosslinked hydrogels were loaded with a drug, protein or dextran during the gel fabrication process as described above. Immediately after cutting the hydrogels, they were transferred to 5 mL of DPBS contained in Sigmacote-treated scintillation vials (to prevent molecular adsorption to the walls of the vial) and simulated with a 20 kHz ultrasonicator using a standard 1/8” microtip (5.4” L × 0.5” dia, titanium alloy, 0.25 lbs.) sonication probe (QSONICA, Newtown, CT) at various ultrasonic amplitudes (%) and durations: amplitudes ranging from 0% to 40% for durations between 1 and 5 min. Previous analyses revealed that 20% US amplitudes corresponded to ultrasonic powers of 0.714 ± 0.02 W and 40% US amplitudes corresponded to 2.09 ± 0.05 W. Immediately after stimulation, 1 mL samples were taken from the scintillation vials for later analysis to quantify the amount of molecular release. In time-course experiments where multiple samples were acquired to plot release vs. time, 1 mL samples were removed from the vials and saved for later analysis, and the remaining 4 mL was removed and discarded. A fresh 5 mL of DPBS was added for the next time point. The concentration of FITC-dextran release was quantified using fluorescence ex/em at 495 nm and 525 nm in a Cytation3 microplate reader (BioTek, Winooski, VT, USA) against a standard curve. The concentrations of mitoxantrone, 5-fluorouracil and irinotecan release samples were quantified by measuring optical absorbance at 610 nm, 350 nm, and 370 nm, respectively, on the plate reader against a standard curve. ELISA was used to measure the concentration of signaling factor release (VEGF and PDGF).

### 4.4. Sequential Release Studies

Alginate hydrogels (1.6 wt%) were crosslinked with 50 mM CaSO_4_ solutions and loaded with irinotecan at 100 µg/gel using methods described in the previous sections. Immediately after cutting the 8-mm-diamter cylindrical hydrogels, they were rinsed for 5 min with 5 mL of DPBS to remove unincorporated irinotecan. Then each gel was soaked with 100 µL of 2 mg/mL 5FU for 30 min to load then with 5FU in a manner that would facilitate rapid 5FU release. After soaking, gels were transferred into new scintillation vials. The experiments began when 5 mL of DPBS was added to the scintillation vials. At 18 h, gels were subjected to ultrasound stimulation at 40% US amplitude for 2 min. Samples were collected at various time points and analyzed for irinotecan and 5FU release using a plate reader as described in the previous section.

### 4.5. Data Representation and Statistical Analysis

All quantitative data presented here are represented by means ± standard deviation. For all statistical analyses, one-way Analysis of Variance (ANOVA) was used with Tukey’s post-hoc tests for multiple comparisons (using Kaleidagraph software, version 4.5.2, Synergy Software, Reading, PA, USA) with p-values of less than 0.05 being our benchmark for statistical significance. *, **, ***, and **** indicates statistical significance of p < 0.05, 0.01, 0.001, and 0.0001 respectively. n.s. indicates no statistical significance. (p > 0.05).

## 5. Conclusions

The studies presented here revealed that the ultrasonic exposures needed to generate statistically significant therapeutic deliveries from calcium-crosslinked hydrogels also generated unacceptably high levels of gel heating and erosion. However, further studies demonstrated that pulsing the ultrasound (i.e., repeated on/off cycles) could reduce gel heating and erosion while producing statistically significant amounts of therapeutic release. This work went on to demonstrate that ultrasound could generate pulsatile chemotherapeutic delivery profiles similar to those shown to enhance melanoma cell destruction. Finally, these studies demonstrated that sequential delivery of dual anticancer agents could enhance melanoma cell destruction and that ultrasonically responsive gels could reproduce similar, sequential anticancer therapeutic delivery profiles.

## Supplementary Materials

The following are available online, Descriptions of melanoma cell survival study methodologies and results (Figure S1) are included in supplementary materials.

## References

[1] Richardson T.P., Peters M.C., Ennett A.B., Mooney D.J. (2001). Polymeric system for dual growth factor delivery. Nat. Biotechnol..

[2] Brudno Y., Ennett-Shepard A.B., Chen R.R., Aizenberg M., Mooney D.J. (2013). Enhancing microvascular formation and vessel maturation through temporal control over multiple pro-angiogenic and pro-maturation factors. Biomaterials.

[3] Mehta M., Schmidt-Bleek K., Duda G.N., Mooney D.J. (2012). Biomaterial delivery of morphogens to mimic the natural healing cascade in bone. Adv. Drug Deliv. Rev..

[4] Spiller K.L., Nassiri S., Witherel C.E., Anfang R.R., Ng J., Nakazawa K.R., Yu T., Vunjak-Novakovic G. (2015). Sequential delivery of immunomodulatory cytokines to facilitate the M1-to-M2 transition of macrophages and enhance vascularization of bone scaffolds. Biomaterials.

[5] Martinez F.O., Gordon S. (2014). The M1 and M2 paradigm of macrophage activation: Time for reassessment. F1000Prime Rep..

[6] Betz O.B., Betz V.M., Nazarian A., Egermann M., Gerstenfeld L.C., Einhorn T.A., Vrahas M.S., Bouxsein M.L., Evans C.H. (2007). Delayed administration of adenoviral BMP-2 vector improves the formation of bone in osseous defects. Gene Ther..

[7] Bertone A.L., Pittmann D.D., Bouxsein M.L., Li J., Clancy B., Seeherman H.J. (2004). Adenoviral-mediated transfer of hBMP-6 gene accelerates osteotomy repair and return of bone mechanical properties. J. Orthop. Res..

[8] Ito T., Tokunaga K., Maruyama H., Kawashima H., Kitahara H., Horikoshi T., Ogose A., Hotta Y., Kuwano R., Katagiri H. (2003). Coxsackievirus and adenovirus receptor (CAR)-positive immature osteoblasts as targets of adenovirus-mediated gene transfer for fracture healing. Gene Ther..

[9] Gerstenfeld L.C., Cullinane D.M., Barnes G.L., Graves D.T., Einhorn T.A. (2003). Fracture healing as a post-natal developmental process: Molecular, spatial, and temporal aspects of its regulation. J. Cell. Biochem..

[10] Kearney C.J., Mooney D.J. (2013). Macroscale delivery systems for molecular and cellular payloads. Nat. Mater..

[11] Cara S., Tannock I.F. (2001). Retreatment of patients with the same chemotherapy: Implications for clinical mechanisms of drug resistance. Ann. Oncol..

[12] Mormont M.C., Levi F. (2003). Cancer chronotherapy: Principles, applications, and perspectives. Cancer.

[13] Mormont M.C., Lévi F. (1997). Circadian-system alterations during cancer processes: A review. Int. J. Cancer.

[14] Goldman A., Majumder B., Dhawan A., Ravi S., Goldman D., Kohandel M., Majumder P.K., Sengupta S. (2015). Temporally sequenced anticancer drugs overcome adaptive resistance by targeting a vulnerable chemotherapy-induced phenotypic transition. Nat. Commun..

[15] Araujo J.C., Mathew P., Armstrong A.J., Braud E.L., Posadas E., Lonberg M., Gallick G.E., Trudel G.C., Paliwal P., Agrawal S. (2012). Dasatinib combined with docetaxel for castration-resistant prostate cancer: Results from a phase 1–2 study. Cancer.

[16] Lee M.J., Ye A.S., Gardino A.K., Heijink A.M., Sorger P.K., MacBeath G., Yaffe M.B. (2012). Sequential application of anticancer drugs enhances cell death by rewiring apoptotic signaling networks. Cell.

[17] Xiao D., Yang D., Guo L., Lu W., Charpentier M., Yan B. (2013). Regulation of carboxylesterase-2 expression by p53 family proteins and enhanced anti-cancer activities among 5-fluorouracil, irinotecan and doxazolidine prodrug. Br. J. Pharmacol..

[18] Wu M.H., Yan B., Humerickhouse R., Dolan M.E. (2002). Irinotecan Activation by Human Carboxylesterases in Colorectal Adenocarcinoma Cells. Clin. Cancer Res..

[19] Lévi F., Zidani R., Misset J.-L. (1997). Randomised multicentre trial of chronotherapy with oxaliplatin, fluorouracil, and folinic acid in metastatic colorectal cancer. Lancet.

[20] Yu T., Huang X., Hu K., Bai J., Wang Z. (2004). Treatment of transplanted adriamycin-resistant ovarian cancers in mice by combination of adriamycin and ultrasound exposure. Ultrason. Sonochem..

[21] Douillard J.Y., Cunningham D., Roth A.D., Navarro M., James R.D., Karasek P., Jandik P., Iveson T., Carmichael J., Alakl M. (2000). Irinotecan combined with fluorouracil compared with fluorouracil alone as first-line treatment for metastatic colorectal cancer: A multicentre randomised trial. Lancet.

[22] Hoare T.R., Kohane D.S. (2008). Hydrogels in drug delivery: Progress and challenges. Polymer.

[23] Alvarez-Lorenzo C., Bromberg L., Concheiro A. (2009). Light-sensitive Intelligent Drug Delivery Systems^†^. Photochem. Photobiol..

[24] Bromberg L.E., Ron E.S. (1998). Temperature-responsive gels and thermogelling polymer matrices for protein and peptide delivery. Adv. Drug Deliv. Rev..

[25] Kennedy S., Bencherif S., Norton D., Weinstock L., Mehta M., Mooney D. (2014). Rapid and Extensive Collapse from Electrically Responsive Macroporous Hydrogels. Adv. Healthc. Mater..

[26] Hoare T., Santamaria J., Goya G.F., Irusta S., Lin D., Lau S., Padera R., Langer R., Kohane D.S. (2009). A Magnetically Triggered Composite Membrane for On-Demand Drug Delivery. Nano Lett..

[27] Hu S.-H., Liu T.-Y., Liu D.-M., Chen S.-Y. (2007). Controlled Pulsatile Drug Release from a Ferrogel by a High-Frequency Magnetic Field. Macromolecules.

[28] Liu T.-Y., Hu S.-H., Liu T.-Y., Liu D.-M., Chen S.-Y. (2006). Magnetic-sensitive behavior of intelligent ferrogels for controlled release of drug. Langmuir.

[29] Hu S.-H., Liu T.-Y., Liu D.-M., Chen S.-Y. (2007). Nano-ferrosponges for controlled drug release. J. Contol. Release.

[30] Zhao X., Kim J., Cezar C.A., Huebsch N., Lee K., Bouhadir K., Mooney D.J. (2011). Active scaffolds for on-demand drug and cell delivery. Proc. Natl. Acad. Sci. USA.

[31] Cezar C.A., Kennedy S.M., Mehta M., Weaver J.C., Gu L., Vandenburgh H., Mooney D.J. (2014). Biphasic Ferrogels for Triggered Drug and Cell Delivery. Adv. Healthc. Mater..

[32] Epstein-Barash H., Orbey G., Polat B.E., Ewoldt R.H., Feshitan J., Langer R., Borden M.A., Kohane D.S. (2010). A microcomposite hydrogel for repeated on-demand ultrasound-triggered drug delivery. Biomaterials.

[33] Huebsch N., Kearney C.J., Zhao X., Kim J., Cezer C.A., Suo Z., Mooney D.J. (2014). Ultrasound-triggered disruption and self-healing of reversibly cross-linked hydrogels for drug delivery and enhanced chemotherapy. Proc. Natl. Acad. Sci. USA.

[34] Wu C.H., Sun M.K., Shieh J., Chen C.S., Huang C.W., Dai C.A., Chang S.W., Chen W.S., Young T.H. (2018). Ultrasound-responsive NIPAM-based hydrogels with tunable profile of controlled release of large molecules. Ultrasonics.

[35] Jiang H., Tovar-Carrillo K., Kobayashi T. (2016). Ultrasound stimulated release of mimosa medicine from cellulose hydrogel matrix. Ultrason. Sonochem..

[36] Miller D.L., Smith N.B., Bailey M.R., Czarnota G.J., Hynynen K., Makin I.R.S. (2012). Overview of Therapeutic Ultrasound Application and Safety Considerations. J. Ultrasound Med..

[37] Mitragotri S., Kost J. (2004). Low-frequency sonophoresis: A review. Adv. Drug Deliv. Rev..

[38] Tezel A., Sens A., Mitragotri S. (2002). Investigations of the role of cavitation in low-frequency sonophoresis using acoustic spectroscopy. J. Pharm. Sci..

[39] Kearney C.J., Skaat H., Kennedy S.M., Hu J., Darnell M., Raimondo T.M., Mooney D.J. (2015). Switchable Release of Entrapped Nanoparticles from Alginate Hydrogels. Adv. Healthc. Mater..

[40] Tolouei A.E., Dulger N., Ghatee R., Kennedy S. (2018). A Magnetically Responsive Biomaterial System for Flexibly Regulating the Duration between Pro- and Anti-Inflammatory Cytokine Deliveries. Adv. Healthc. Mater..

[41] Kennedy S., Roco C., Deleris A., Spoerri P., Cezar C., Weaver J., Vandenburgh H., Mooney D. (2018). Improved magnetic regulation of delivery profiles from ferrogels. Biomaterials.

[42] Kennedy S., Hu J., Kearney C., Skaat H., Gu L., Gentili M., Vandenburgh H., Mooney D. (2016). Sequential release of nanoparticle payloads from ultrasonically burstable capsules. Biomaterials.

[43] Emi T.T., Barnes T., Orton E., Reisch A., Tolouei A.E., Madani S.Z.M., Kennedy S.M. (2018). Pulsatile Chemotherapeutic Delivery Profiles Using Magnetically Responsive Hydrogels. ACS Biomater. Sci. Eng..

[44] Burgess S.E., Silverman R.H., Coleman D.J., Yablonski M.E., Lizzi F.I., Driller J., Rosado A., Dennis P.H. (1986). Treatment of glaucoma with high-intensity focused ultrasound. Ophthalmology.

[45] Lepock J.R. (2005). How do cells respond to their thermal environment?. Int. J. Hyperth..

[46] Bischof J.C., He X. (2005). Thermal stability of proteins. Ann. N. Y. Acad. Sci..

[47] Berrada M., Yang Z., Lehnert S. (2002). Tumor treatment by sustained intratumoral release of 5-fluorouracil: Effects of drug alone and in combined treatments. Int. J. Radiat. Oncol. Biol. Phys..

