# Supplementary material for: Ultrasonic Generation of Pulsatile and Sequential Therapeutic Delivery Profiles from Calcium-Crosslinked Alginate Hydrogels
Source: Molecules. 2019 Mar 16;24(6):1048. doi: 10.3390/molecules24061048 (PMC6470886; doi:10.3390/molecules24061048)
Supplement: Supplementary file 1 [file molecules-24-01048-s001.pdf]

Supplementary Materials

S.1. Melanoma Cell Culture and Maintenance

B16F10 mouse melanoma cells were cultured in DMEM with 10% FBS and 1% penicillin-streptomycin at 37ºC in 5% CO_2. B16F10s were routinely split to avoid confluence over 70% (roughly every other day) by trypsinizing (5 min with 0.25% w/v trypsin and 0.5 mM EDTA), collecting, centrifuging, washing, and re-seeding on 75 cm^2 flasks.

S.2. Exposure of Melanoma Cells to Sequential 5FU and Irinotecan Deliveries

The effect of sequential 5-fluorouracil and irinotecan exposure was compared with delivering 5-fluorouracil (5FU) and irinotecan to B16F10 mouse melanoma cells in vitro on an xCELLigence real-time cell index system. B16F10s were seeded at 500 cells per cm^2 on 16-well xCELLigence e-plates and allowed to grow in DMEM for 36 h. Then, B16F10s were exposed to various combinations of 5-fluorouracil and irinotecan (Figure S1a, schedules S1 to S4) for 36 h. For delivery schedules that involved changing the drug vs. time (i.e., schedules S3 and S4 for sequential deliveries of 5FU and irinotecan), cells were rinsed 3 times with fresh DMEM immediately after removing the first drug (at the 18-h mark) as to remove any residual drug before adding the other drug. Real-time cell index data (indicating cell population levels) were collected using xCELLigence software for analysis and plotting. Cell index values were normalized and set to 1 at time 0 when treatment began. Each condition S1 through S4 was repeated in 4 separate wells (N = 4) to compute means and standard deviation. Results of this study are included in Figure S1, bellow. Note that a control condition is not included (i.e., one where no chemotherapeutic is delivered). This was not included because cell index values saturated the xCELLigence system when no anticancer agent was present to impede B16F10 growth (i.e., B16F10s reached confluency during the experiment, saturating the cell index signal).

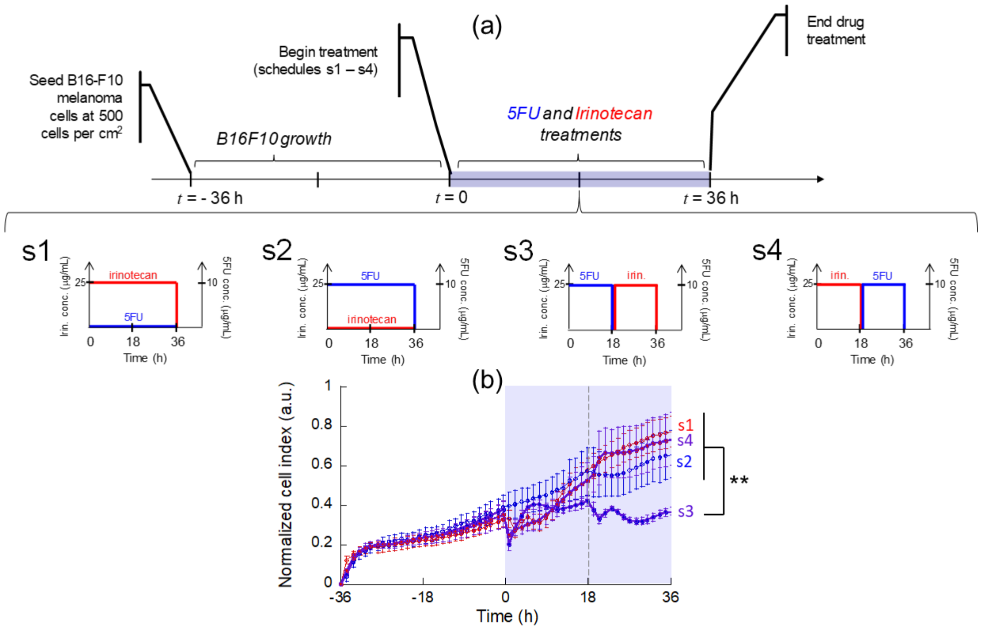

Figure S1: 5-fluorouracil and irinotecan delivery sequence had an impact on melanoma cell survival. (a) A timeline describing the in vitro cytotoxicity experiment where B16F10 mouse melanoma cells were seeded and allowed to grow for 36 h before treatment and exposed to several treatment schedules s1-s4 for 36 h. (b) Normalized cell index (B16-F10 cell population) vs. time when exposed to s1 (red curve), s2 (blue curve), s3 (purple curve, blue marker), s4 (purple curve, red marker). ** indicates a statistically significant difference with $p < 0.01$. N = 4.

S.3. Erosion test of gel formulated for sequential release before and after 18 h

To observe the degree of gel erosion caused during experiments designed to demonstrate sequential 5FU and irinotecan release, alginate hydrogels were formed similar to those in the 5FU-irinotecan release studies (1.6 wt% alginate crosslinked with 50 mM calcium, see Section 4.4). However, to enable visual inspection of hydrogel erosion vs. time, gels were loaded with mitoxantrone (at 40 μg/gel), a blue chemotherapeutic (note that 5FU and irinotecan are clear to the naked eye). Gels were dropped in DPBS and allowed to sit for 18 h (exchanging media according to the time course experiments featuring sequential release of 5FU and irinotecan: at 2 and 18 h). A 40% amplitude ultrasonic signal was applied for 2 min starting at 18 h and the media was exchanged immediately following stimulation. Gels were left to sit for an additional 18 h to round out the 36-h experiment. Photographs of the gel at different stages of this 36-h experiment can be seen in Figure S2.

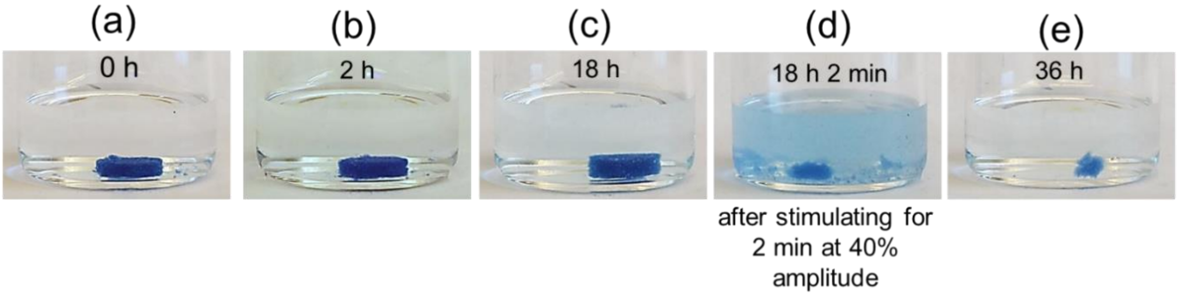

Figure S2: Gels remained intact prior to ultrasonic stimulation but significantly eroded after stimulation. Photographs of a mitoxantrone-loaded gel (for visually staining the gel blue) at the beginning of the experiment (a), after the first 2 h (b), at 18 h just prior to ultrasonic stimulation (c), immediately following 2 min of ultrasonic stimulation at 40% amplitude (d), and at the end of the 36-h experiment (e).
